# Lactate and procalcitonin combined with qSOFA score for the evaluation of disease severity and prognosis in ICU patients with sepsis: a retrospective study

**DOI:** 10.3389/fmed.2025.1722546

**Published:** 2025-12-11

**Authors:** Qingzhao Tan, Huaying Chen, Xianjin Zhang

**Affiliations:** Department of Emergency, Zhongshan Hospital, Xiamen University, Xiamen, Fujian, China

**Keywords:** qSOFA, lactate, procalcitonin, dynamic monitoring, sepsis, prognosis

## Abstract

**Background:**

Sepsis remains a leading cause of mortality among Intensive Care Unit (ICU) patients, and early prognostic assessment is essential for improving outcomes. Although the quick Sequential Organ Failure Assessment (qSOFA) score is widely used for bedside risk stratification, its standalone predictive performance is limited. Integrating biochemical markers such as lactate and procalcitonin (PCT) may improve prognostic accuracy. This study was designed to evaluate the combined value of qSOFA, lactate, and PCT in assessing disease severity and mortality risk, and to clarify the prognostic utility of dynamic monitoring at multiple time points.

**Methods:**

This retrospective study included 128 ICU patients with sepsis, categorized into qSOFA < 2 (*n* = 57) and qSOFA ≥ 2 (*n* = 71) groups. Lactate and PCT levels were recorded at ICU admission (T0), 24 h (T24), and 72 h (T72). ICU mortality, 28-day mortality, ICU length of stay, and multiple organ failure rates were analyzed. Multivariate logistic regression was performed with adjustment for age, hypertension, and SOFA score. Receiver operating characteristic (ROC) curves were used to compare the predictive performance of qSOFA alone and in combination with the biomarkers.

**Results:**

Patients with qSOFA ≥ 2 had significantly higher ICU and 28-day mortality (*P* < 0.05) and exhibited consistently elevated lactate and PCT levels at all three time points (*P* < 0.01). qSOFA, lactate, and PCT were independent predictors of both ICU and 28-day mortality (*P* < 0.05). The combined model demonstrated superior discrimination, with AUCs of 0.79 for ICU mortality and 0.81 for 28-day mortality, outperforming qSOFA alone or qSOFA paired with a single biomarker.

**Conclusion:**

A qSOFA score ≥ 2 identifies patients at higher risk of mortality. Dynamic monitoring of lactate and PCT significantly enhances early prognostic evaluation, and their combined use with qSOFA provides improved risk stratification for ICU patients with sepsis.

## 1 Introduction

Sepsis remains a major challenge in critical care, frequently leading to multiple organ dysfunction and high mortality. Global epidemiological data indicate a continuing rise in deaths due to severe infections and septic shock among hospitalized patients (1). The Sepsis 3.0 definition introduced the Sequential Organ Failure Assessment (SOFA) score and the quick SOFA (qSOFA) score to aid in the early identification of disease severity and to guide timely clinical intervention (2, 3). While qSOFA offers a simple bedside tool for rapid screening of high-risk patients, its standalone sensitivity has been repeatedly questioned, and its performance varies across clinical settings (4). To address these limitations, integrating additional biomarkers has been proposed to improve the accuracy of early risk stratification. Among these, serum lactate and procalcitonin (PCT) have emerged as critical indicators—lactate reflecting tissue hypoperfusion and metabolic stress, and PCT signaling the host’s response to bacterial infection. Assessing these markers in conjunction with qSOFA may enhance prognostic evaluation in septic patients (5, 6).

In critically ill patients, physiological and immunological parameters can change rapidly, necessitating continuous, precise monitoring. Although several studies support the value of serial (multi-time-point) assessments of lactate and PCT, the prognostic implications of measurements taken at different intervals (e.g., early vs. delayed changes) remain inconsistent across the literature, and the optimal timing for repeated biomarker evaluation has not been clearly established (7). Furthermore, in resource-limited settings, challenges in obtaining complete serial data limit the validation of combined assessments using qSOFA, lactate, and PCT (8). To fill these gaps, we conducted a retrospective study to investigate whether the combination of qSOFA, lactate, and PCT enhances early prediction of mortality risk in Intensive Care Unit (ICU) patients with sepsis. This study evaluates the predictive value of these indicators—individually and in combination—using standardized time points (within 6, 24 ± 4, and 72 ± 8 h after ICU admission). Using multivariate modeling and ROC curve analysis, we aim to determine whether combining a rapid bedside score with routinely available biomarkers improves risk stratification and early prognostic assessment, thereby supporting more efficient and targeted clinical decision-making in sepsis management.

## 2 Materials and methods

### 2.1 Study design

This was a retrospective, single-center study conducted at the Department of Critical Care Medicine, Zhongshan Hospital of Xiamen University, covering the period from January 2023 to December 2024. The study protocol was approved by the Zhongshan Hospital Medical Ethics Committee (Approval No. 2019007). Data were retrieved from the hospital’s electronic medical records and laboratory databases by searching for sepsis-related diagnostic terms and ICU admissions. All data were anonymized prior to analysis.

### 2.2 Study subjects

#### 2.2.1 Inclusion criteria

Patients were eligible for inclusion if they had a confirmed diagnosis of sepsis or septic shock based on Sepsis 3.0 criteria, characterized by infection-induced organ dysfunction with a SOFA score ≥ 2. Additional inclusion requirements were age ≥ 18 years, hospitalization in the Department of Critical Care Medicine with receipt of ICU treatment, an admission date between January 2023 and December 2024, and availability of lactate and PCT measurements within the first 72 h of ICU admission. For the present analysis, only patients with complete data at all three predefined time points were retained.

#### 2.2.2 Exclusion criteria

**FIGURE 1:**
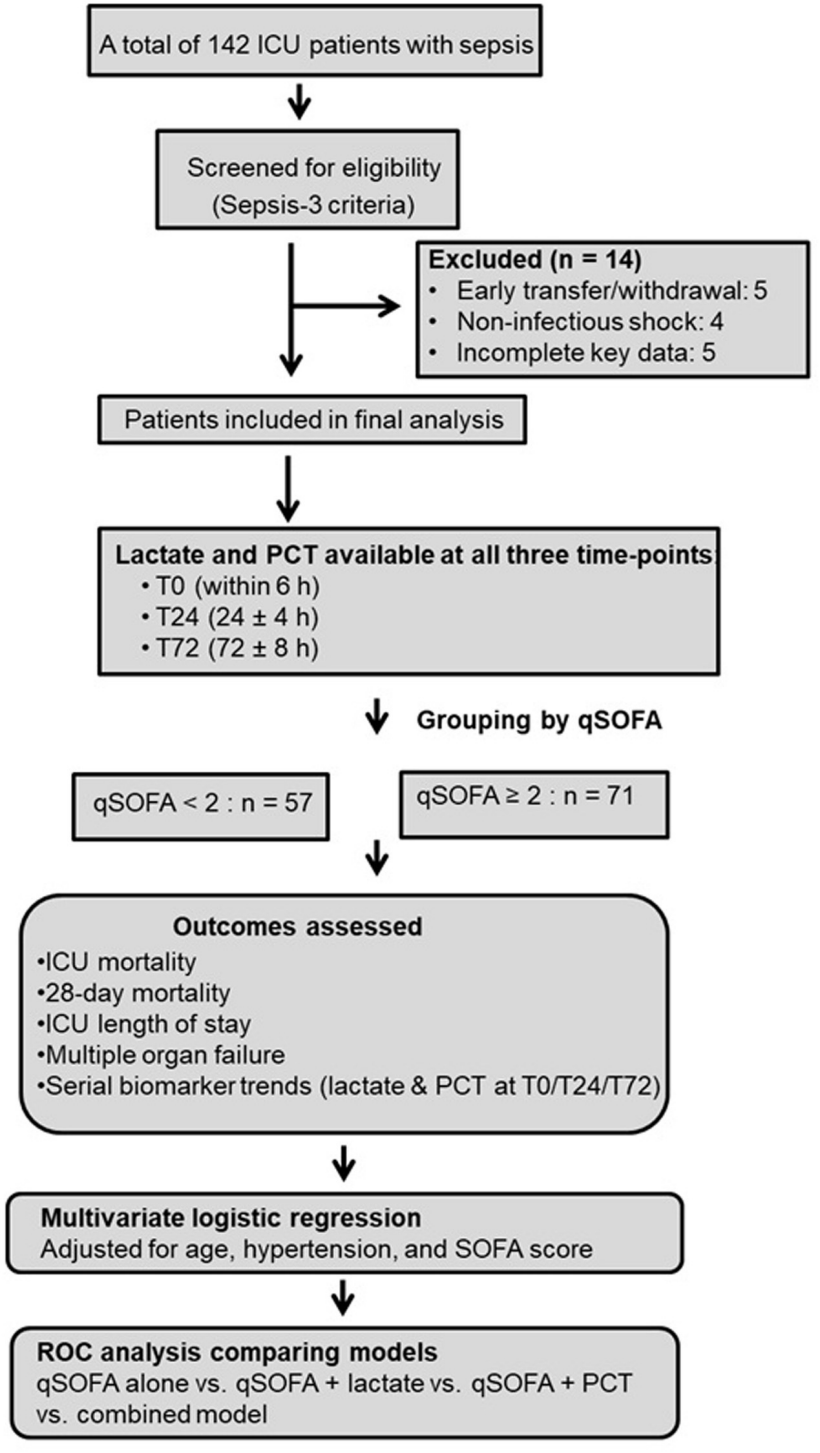
Study flow diagram. A total of 142 patients were screened; 14 were excluded. The final cohort (*n* = 128) had complete lactate and procalcitonin (PCT) measurements at T0, T24, and T72 and was divided into survival and non-survival groups for analysis.

Patients were excluded if they had non-infectious shock or organ dysfunction unrelated to sepsis, terminal malignancy with an expected survival of less than 3 months, pregnancy, or if they were transferred out of the ICU or withdrew from active treatment within 24 h of admission. Patients with severe immunosuppression (e.g., advanced AIDS or post-transplant immunosuppression) were excluded only when essential clinical or laboratory data were incomplete. Individuals were also excluded if they had missing key variables required for outcome or multivariable analyses. A total of 142 patients initially met Sepsis 3.0 criteria. Of these, 14 patients were excluded (five due to early transfer or withdrawal, four due to non-infectious shock, and five due to incomplete key data). The final study population comprised 128 patients with complete lactate and PCT measurements at all three standardized time points: within 6 (T0), 24 ± 4 (T24), and 72 ± 8 h (T72) after ICU admission. The screening, exclusion, and final inclusion of patients are summarized in the study flow diagram (Figure 1).

### 2.3 Data collection and variable definitions

#### 2.3.1 Demographic and clinical information

Age and sex were obtained from electronic records. Comorbidities, including hypertension, diabetes, COPD, and chronic renal insufficiency, were identified from documented medical histories. Infection sites (lung, abdomen, urinary tract, bloodstream) were classified based on admission diagnoses. Microbiological culture results, when available, were recorded. The primary reason for ICU admission was extracted from physician notes.

#### 2.3.2 Laboratory indicators and time points

Serum lactate levels (mmol/L) were measured by blood gas or biochemical analyzers. PCT levels (ng/mL) were measured by electrochemiluminescence or chemiluminescence immunoassay. For all patients, lactate and PCT values were extracted at three predefined time points: T0 (within 6 h of ICU admission), T24 (24 ± 4 h), and T72 (72 ± 8 h). Only values within these windows were included; no imputation or extrapolation of missing biomarker values was performed. Because this was a retrospective study, serial measurements represent absolute values, and relative changes (e.g., lactate clearance, Δ-PCT) could not be calculated.

### 2.4 Scoring indicators

The qSOFA score included systolic blood pressure ≤ 100 mmHg, respiratory rate ≥ 22/min, and altered mental status (GCS < 15), assessed within the first 24 h after ICU admission. Scores ranged from 0 to 3. SOFA and APACHE II scores were extracted from admission records and used as covariates (9–11). Two researchers independently verified the scores, and discrepancies were resolved by a third reviewer.

### 2.5 Prognostic indicators

ICU mortality was defined as death during ICU hospitalization. Twenty-eight-day mortality was determined based on survival status 28 days after sepsis diagnosis. ICU length of stay was calculated in days. Multiple organ failure was defined as dysfunction of two or more organ systems, based on laboratory and clinical documentation.

### 2.6 Grouping strategy

Patients were grouped according to qSOFA score into qSOFA < 2 and qSOFA ≥ 2. Lactate and PCT values at T0, T24, and T72 were used to analyze serial trends and their prognostic significance. This grouping allowed evaluation of the incremental value of combining a bedside clinical score with repeated biomarker measurements.

### 2.7 Statistical analysis

Statistical analyses were performed using SPSS 26.0. Continuous variables were reported as mean ± standard deviation (SD) or median (IQR) based on Shapiro–Wilk normality testing. Between-group comparisons used the independent-samples *t*-test or Mann–Whitney *U*-test. Categorical variables were analyzed with the χ^2^-test or Fisher’s exact test when expected frequencies were <5. Serial changes in lactate and PCT were evaluated using repeated-measures ANOVA, with “time” (T0, T24, T72) as the within-subject factor and “group” as the between-subject factor. When significant time × group interactions were found, Bonferroni-adjusted *post hoc* comparisons were performed to control for multiple testing. For multivariate analysis, binary logistic regression models were constructed for ICU and 28-day mortality at each time point. Independent variables included qSOFA category, lactate (continuous), PCT (continuous), age, hypertension, and SOFA score. The number of predictors met the ≥10 events-per-variable rule to avoid overfitting. A backward stepwise (likelihood ratio) method was used for model selection. Odds ratios (ORs) with 95% confidence intervals (CIs) were reported. Multicollinearity among qSOFA, SOFA, lactate, and PCT was assessed using variance inflation factors (VIF), all <2, indicating no significant collinearity. Predictive accuracy was evaluated using the area under the ROC curve (AUC) for qSOFA alone, qSOFA + lactate, qSOFA + PCT, and qSOFA + lactate + PCT. The optimal cutoff was derived from the Youden index, and sensitivity, specificity, PPV, and NPV were calculated. AUC values are presented with 95% CIs.

## 3 Results

### 3.1 Baseline characteristics and grouping

Among the 128 ICU patients included in the study, 57 were categorized into the qSOFA < 2 group and 71 into the qSOFA ≥ 2 group. Baseline demographic variables, comorbidities (hypertension, diabetes), and infection sources (pulmonary, abdominal, urinary, bloodstream) showed no statistically significant differences between groups (*P* > 0.05). However, both SOFA and APACHE II scores were significantly higher in the qSOFA ≥ 2 group compared with the qSOFA < 2 group (*P* < 0.001), indicating greater initial illness severity (Table 1).

**TABLE 1:** Baseline characteristics of ICU patients stratified by quick Sequential Organ Failure Assessment (qSOFA) score.

Variable	qSOFA < 2 (*n* = 57)	qSOFA ≥ 2 (*n* = 71)	Test value	*P*-value
Age (years), mean ± SD	53.47 ± 7.82	58.93 ± 8.72	*t* = 3.084	0.003
Sex (male), *n* (%)	33 (57.89%)	45 (63.38%)	χ^2^ = 0.462	0.498
Hypertension, *n* (%)	21 (36.84%)	31 (43.66%)	χ^2^ = 0.563	0.452
Diabetes, *n* (%)	17 (29.82%)	26 (36.62%)	χ^2^ = 0.662	0.416
Pulmonary infection, *n* (%)	25 (43.86%)	37 (52.11%)	χ^2^ = 1.217	0.271
Abdominal infection, *n* (%)	13 (22.81%)	20 (28.17%)	χ^2^ = 0.475	0.496
Urinary infection, *n* (%)	9 (15.79%)	11 (15.49%)	χ^2^ = 0.007	0.955
Bloodstream infection, *n* (%)	7 (12.28%)	13 (18.31%)	χ^2^ = 1.023	0.313
Positive microbiological culture	31 (54.39%)	43 (60.56%)	χ^2^ = 0.483	0.487
SOFA score, mean ± SD	3.54 ± 0.51	6.33 ± 0.79	*t* = 19.642	<0.001
APACHE II score, mean ± SD	13.48 ± 1.75	18.69 ± 2.52	*t* = 12.116	<0.001

Continuous variables were compared using independent-samples *t*-tests; categorical variables using χ^2^-tests.

### 3.2 Serum lactate and PCT levels in qSOFA groups

Repeated-measures ANOVA revealed significant time × group interaction effects for both serum lactate and PCT (interaction *P* < 0.05). *Post hoc* comparisons with Bonferroni adjustment showed that serum lactate and PCT concentrations were significantly higher in the qSOFA ≥ 2 group at T0, T24, and T72 (all *P* < 0.01 after correction) (Table 2). The temporal decline in both biomarkers was more pronounced in the qSOFA < 2 group, consistent with the interaction effect.

**TABLE 2:** Comparison of lactate and procalcitonin (PCT) at each time point in quick Sequential Organ Failure Assessment (qSOFA) groups.

Variable	qSOFA < 2 (mean ± SD)	qSOFA ≥ 2 (mean ± SD)	*t*-value	*P*-value
**Lactate (mmol/L)**				
T0	2.13 ± 0.31	2.82 ± 0.41	3.182	0.002
T24	1.94 ± 0.27	2.51 ± 0.34	4.037	0.001
T72	1.58 ± 0.22	2.14 ± 0.29	3.713	<0.001
Interaction (time × group)	–	–	*F* = 6.139	0.014
**PCT (ng/mL)**				
T0	2.47 ± 0.37	3.52 ± 0.54	5.204	<0.001
T24	1.79 ± 0.27	3.01 ± 0.44	6.106	<0.001
T72	1.16 ± 0.17	2.36 ± 0.33	7.457	<0.001
Interaction (time × group)	–	–	*F* = 5.297	0.021

Repeated-measures ANOVA used for interaction effects. Pairwise comparisons performed using Bonferroni-adjusted *t*-tests.

### 3.3 Clinical outcome comparison

Patients in the qSOFA ≥ 2 group had higher ICU mortality (30.99% vs. 12.28%, *P* = 0.011), higher 28-day mortality (39.44% vs. 15.79%, *P* = 0.002), and a greater incidence of multiple organ failure (35.21% vs. 19.30%, *P* = 0.039). ICU length of stay was also significantly longer in the qSOFA ≥ 2 group (14.92 ± 2.01 vs. 10.87 ± 1.32 days, *P* < 0.001) (Table 3).

**TABLE 3:** Clinical outcomes in qSOFA < 2 and qSOFA ≥ 2 groups.

Outcome	qSOFA < 2 (*n* = 57)	qSOFA ≥ 2 (*n* = 71)	Test value	*P*-value
ICU mortality, *n* (%)	7 (12.28%)	22 (30.99%)	χ^2^ = 6.402	0.011
28-day mortality, *n* (%)	9 (15.79%)	28 (39.44%)	χ^2^ = 9.984	0.002
ICU length of stay (days), mean ± SD	10.87 ± 1.32	14.92 ± 2.01	*t* = 8.233	<0.001
Multiple organ failure, *n* (%)	11 (19.30%)	25 (35.21%)	χ^2^ = 4.241	0.039

χ^2^-tests or Fisher’s exact test for categorical variables; *t*-tests for continuous variables.

### 3.4 Serial changes in biomarkers among survivors and non-survivors

**FIGURE 2:**
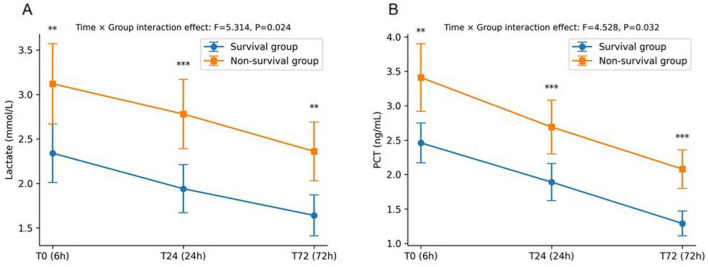
Dynamic trends of lactate and procalcitonin (PCT) in survivors and non-survivors. (A) Dynamic changes in serum lactate at ICU admission (T0), 24 h (T24), and 72 h (T72) in the survival and non-survival groups. Non-survivors demonstrated significantly higher lactate values at all three time points. Repeated-measures ANOVA showed a significant time × group interaction (*F* = 5.314, *P* = 0.024). (B) Dynamic changes in PCT levels at T0, T24, and T72. PCT concentrations remained consistently higher in non-survivors, with significant differences at each time point. Repeated-measures ANOVA revealed a significant time × group interaction (*F* = 4.528, *P* = 0.032). Error bars represent standard deviations. **P < 0.01, ***P < 0.001 (Bonferroni-adjusted *post hoc* comparisons).

Comparison of biomarker trends by survival status showed that survivors had consistently lower serum lactate and PCT values at T0, T24, and T72, with significant differences observed at each time point (*P* < 0.01 or *P* < 0.001). Repeated-measures ANOVA confirmed significant time × survival status interaction effects for both biomarkers, including lactate (*F* = 5.314, *P* = 0.024) and PCT (*F* = 4.528, *P* = 0.032). Bonferroni-adjusted post-hoc tests further verified significant pairwise differences between survivors and non-survivors at each time point (Figures 2A, B).

### 3.5 Multivariate regression and combined predictive value

Logistic regression analyses performed at T0, T24, and T72 showed that qSOFA ≥ 2, higher serum lactate, and higher PCT were independent predictors of both ICU and 28-day mortality, after adjustment for age, hypertension, and SOFA score (all *P* < 0.05). The odds ratios for qSOFA ≥ 2 increased progressively from T0 to T72, and Nagelkerke R^2^ values increased across time points, indicating progressively stronger model performance with later time-point measurements (Tables 4, 5).

**TABLE 4:** Multivariate logistic regression for ICU mortality.

Variable	Model T0 OR (95% CI), *p*	Model T24 OR (95% CI), *p*	Model T72 OR (95% CI), *p*
qSOFA ≥ 2	2.17 (1.10–3.38), 0.013	2.58 (1.24–4.12), 0.008	2.91 (1.46–5.03), 0.002
Lactate (mmol/L)*	1.34 (1.02–1.74), 0.039	1.59 (1.20–2.04), 0.002	1.68 (1.24–2.27), 0.001
PCT (ng/mL)*	1.38 (1.05–1.85), 0.022	1.49 (1.10–2.11), 0.015	1.81 (1.26–2.49), 0.002
Age (years)	1.04 (1.01–1.07), 0.017	1.04 (1.01–1.07), 0.012	1.05 (1.02–1.08), 0.007
Hypertension	1.35 (0.81–2.29), 0.242	1.28 (0.79–2.16), 0.304	1.47 (0.87–2.61), 0.153
SOFA score	1.31 (1.09–1.63), 0.029	1.53 (1.17–1.96), 0.003	1.62 (1.21–2.08), 0.001
−2 Log likelihood	118.476	113.529	107.638
Nagelkerke R^2^	0.328	0.392	0.457

*Lactate and procalcitonin (PCT) treated as continuous variables in all models. All models adjusted for age, hypertension, and Sequential Organ Failure Assessment (SOFA) score.

**TABLE 5:** Multivariate logistic regression for 28-day mortality.

Variable	Model T0 OR (95% CI), *p*	Model T24 OR (95% CI), *p*	Model T72 OR (95% CI), *p*
qSOFA ≥ 2	2.35 (1.18–3.57), 0.013	2.77 (1.43–4.16), 0.004	3.09 (1.54–4.98), 0.002
Lactate (mmol/L)*	1.42 (1.05–1.95), 0.027	1.62 (1.21–2.08), 0.003	1.75 (1.29–2.22), 0.001
PCT (ng/mL)*	1.31 (1.02–1.77), 0.038	1.46 (1.09–2.09), 0.016	1.82 (1.26–2.53), 0.003
Age (years)	1.03 (1.00–1.06), 0.044	1.04 (1.01–1.07), 0.012	1.05 (1.02–1.08), 0.006
Hypertension	1.27 (0.74–2.27), 0.396	1.39 (0.81–2.45), 0.241	1.53 (0.83–2.79), 0.184
SOFA score	1.36 (1.08–1.78), 0.019	1.51 (1.17–2.01), 0.004	1.68 (1.24–2.12), 0.001
−2 Log likelihood	112.492	106.531	100.678
Nagelkerke R^2^	0.291	0.349	0.417

*Lactate and procalcitonin (PCT) treated as continuous variables. All models adjusted for age, hypertension, and Sequential Organ Failure Assessment (SOFA) score.

### 3.6 ROC curve comparison of predictive models

**FIGURE 3:**
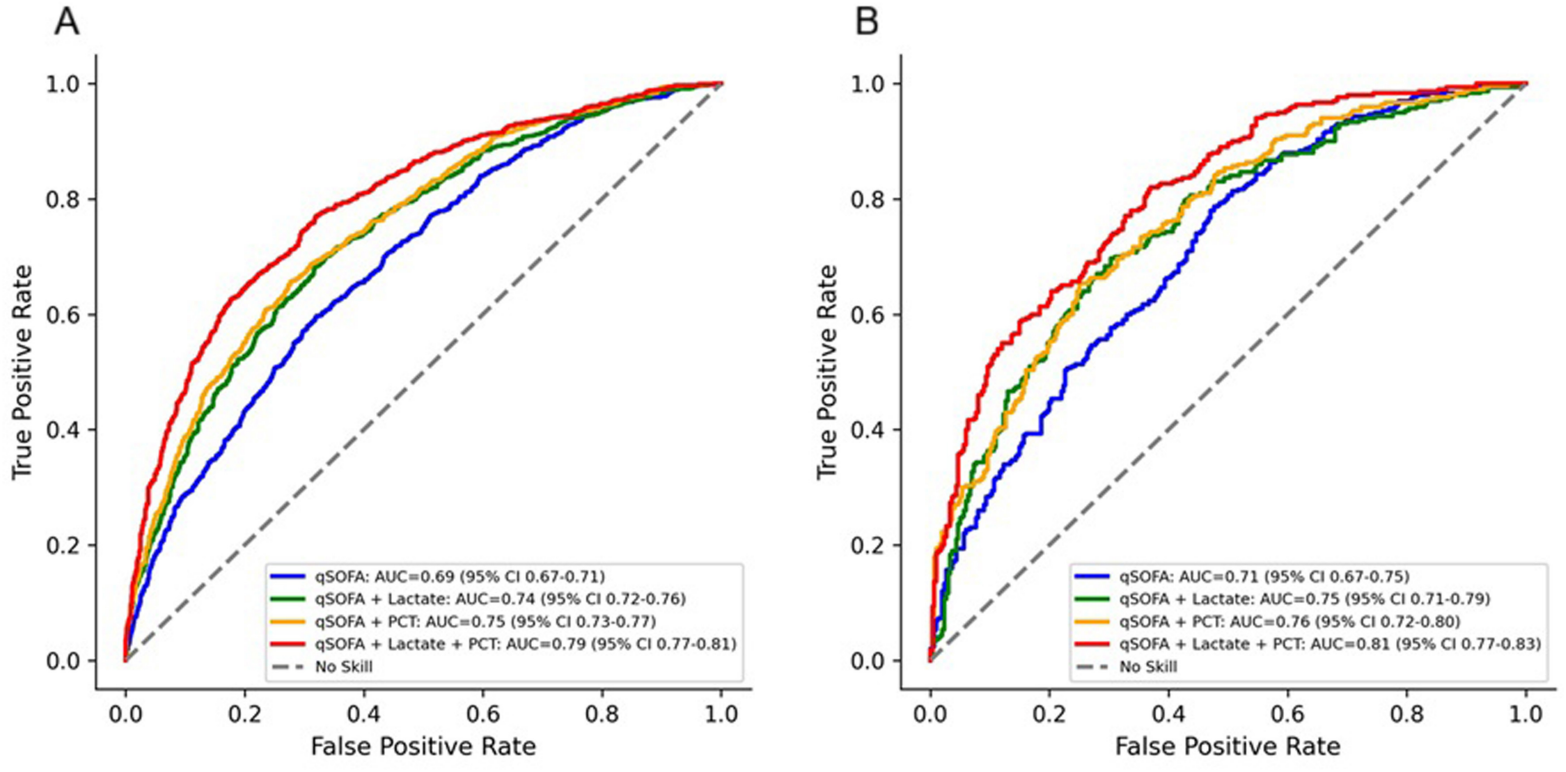
Receiver operating characteristic (ROC) curves for mortality prediction. (A) ROC curves for ICU mortality using qSOFA, qSOFA + lactate, qSOFA + PCT, and the combined model. (B) ROC curves for 28-day mortality using the same models. The AUCs (95% CI) for ICU mortality were: qSOFA 0.69 (0.67–0.71), qSOFA + lactate 0.74 (0.72–0.76), qSOFA + PCT 0.75 (0.73–0.77), and the combined model 0.79 (0.77–0.81). For 28-day mortality, the AUCs (95% CI) were: qSOFA 0.71 (0.67–0.75), qSOFA + lactate 0.75 (0.71–0.79), qSOFA + PCT 0.76 (0.72–0.80), and the combined model 0.81 (0.77–0.83).

Receiver operating characteristic analysis showed that the combined model incorporating qSOFA, lactate, and PCT had the highest predictive accuracy for both ICU mortality (AUC = 0.79, 95% CI 0.77–0.81) and 28-day mortality (AUC = 0.81, 95% CI 0.77–0.83). The predictive performance of qSOFA alone was lower, with AUCs of 0.69 (95% CI 0.67–0.71) for ICU mortality and 0.71 (95% CI 0.67–0.75) for 28-day mortality. Adding a single biomarker improved discrimination, with AUCs of 0.74 (95% CI 0.72–0.76) for qSOFA + lactate and 0.75 (95% CI 0.73–0.77) for qSOFA + PCT for ICU mortality. A similar pattern was observed for 28-day mortality, where qSOFA + lactate achieved 0.75 (95% CI 0.71–0.79) and qSOFA + PCT achieved 0.76 (95% CI 0.72–0.80) (Figures 3A, B). These findings confirm that integrating qSOFA with both biomarkers yields the strongest predictive performance across all models.

## 4 Discussion

The qSOFA score provides a rapid bedside tool for the initial assessment of sepsis severity, relying on systolic blood pressure, respiratory rate, and mental status (12). In this study, patients with qSOFA ≥ 2 demonstrated significantly higher ICU and 28-day mortality, and qSOFA remained an independent predictor of adverse outcomes after multivariable adjustment, underscoring its clinical value in early severity assessment. Although qSOFA is widely incorporated into Sepsis 3.0, its diagnostic sensitivity has been questioned (13). Our findings support its usefulness specifically within an ICU population, where physiological instability is more pronounced and rapid bedside stratification remains essential.

Compared with more comprehensive scoring systems such as SOFA or APACHE II, qSOFA requires fewer parameters and is easier to apply repeatedly at the bedside (14). The present study demonstrates that its prognostic ability improves substantially when combined with serum lactate and PCT, suggesting that qSOFA alone may be insufficient for dynamic risk evaluation, but its performance strengthens when paired with physiologic and infection-related biomarkers. This is particularly relevant in clinical environments where the frequency of comprehensive scoring may be limited by staffing, workflow, or laboratory constraints.

Serum lactate and PCT provide complementary physiologic information. Persistent lactate elevation reflects impaired tissue perfusion and cellular metabolic stress, while increased PCT indicates a sustained systemic inflammatory response driven by bacterial infection (15). In this study, higher lactate and PCT values at T0, T24, and T72 were consistently associated with non-survivors, emphasizing the prognostic value of serial biomarker assessment. While absolute values were used in the present analysis, changes over time—such as lactate clearance or reductions in PCT—may further enhance risk stratification and should be explored in future studies. Elevated or non-declining biomarkers across 24–72 h may reflect persistent infection, inadequate source control, or treatment response failure. The combined model integrating qSOFA, lactate, and PCT demonstrated superior predictive performance for both ICU and 28-day mortality. These parameters contribute different domains of physiologic and pathobiologic information: qSOFA captures early organ dysfunction, lactate reflects global hypoperfusion, and PCT quantifies infection-driven inflammatory burden (16). The ROC analysis confirmed that the integrated model achieved higher AUC values than models using qSOFA alone or qSOFA with a single biomarker, aligning with current evidence supporting multimodal prognostic strategies in sepsis (17). This complementary mechanism provides a plausible explanation for why combining these indicators improves predictive accuracy. The practicality of this combined approach is another important consideration. qSOFA requires no laboratory testing, lactate measurement is routine in most ICUs, and PCT availability continues to expand. This makes the combined model feasible even in settings with limited diagnostic infrastructure, where early prognostic information can support timely decisions regarding monitoring intensity, antimicrobial optimization, and hemodynamic support (18). The multitime-point design (T0–T72) also reflects real-world ICU practice, where clinicians frequently reassess patients as treatment progresses.

Several important limitations should be acknowledged. First, the retrospective single-center design introduces potential selection bias, and the findings may not be fully generalizable to other ICUs with different patient populations or treatment practices. Second, although exclusion criteria were clearly defined, missing data may not have occurred at random, and the absence of a detailed missing-data mechanism is a limitation. Third, the study did not evaluate relative biomarker changes such as lactate clearance or Δ-PCT, which are recommended in dynamic prognostic models. Fourth, although multicollinearity among qSOFA, SOFA, lactate, and PCT was formally assessed using variance inflation factors and no major problems were detected, residual collinearity cannot be completely excluded and may still influence the precision of multivariable estimates. Fifth, the regression model selection process and covariate justification, although standardized, may still carry a risk of overfitting given the limited sample size, particularly for subgroup analyses. Sixth, definitions of organ-specific dysfunction and SOFA subscores were not analyzed separately, limiting reproducibility for the organ failure component. Seventh, infection sources were reported, but detailed subgroup analyses were not feasible due to the modest sample size, and any statements implying subgroup differences have been removed to avoid unsupported claims. Additionally, APACHE II and SOFA scores were included descriptively but not fully incorporated into comparative predictive modeling, limiting interpretation of their incremental value. Finally, as with most retrospective studies, decision-making such as biomarker testing frequency, antimicrobial adjustments, and timing of interventions was not standardized, which may introduce uncontrolled confounding. Future research should include prospective multicenter cohorts with standardized biomarker monitoring protocols, formal evaluation of dynamic indicators such as lactate clearance, and integration of these parameters into sepsis bundles or electronic decision-support tools, which may help clarify the clinical role of this combined prognostic strategy across diverse ICU settings.

## 5 Conclusion

This study demonstrates that the qSOFA score, when combined with dynamic monitoring of lactate and procalcitonin, significantly improves early risk assessment and prognostic prediction in ICU patients with sepsis. While qSOFA provides rapid bedside evaluation, lactate and PCT add essential biochemical information related to tissue perfusion and infection status, enhancing the accuracy of mortality prediction. The integrated model offers better discrimination for ICU and 28-day mortality than qSOFA alone and supports a practical, multidimensional approach to sepsis assessment. In resource-limited settings, a stepwise strategy starting with qSOFA and lactate and adding PCT for high-risk cases may be particularly feasible. Further large, multicenter studies are needed to validate this model and optimize its application across diverse sepsis populations.

## Data Availability

The raw data supporting the conclusions of this article will be made available by the authors, without undue reservation. Requests to access these datasets should be directed to Xianjin Zhang, zsyyjz2120@xmu.edu.cn.
